# TSH enhances neurite outgrowth

**DOI:** 10.3389/fendo.2024.1463964

**Published:** 2024-10-17

**Authors:** Maryam Mansoori, Rauf Latif, Syed A. Morshed, Mone Zaidi, Terry F. Davies

**Affiliations:** ^1^ Thyroid Research Unit, Icahn School of Medicine at Mount Sinai, New York, NY, United States; ^2^ Department of Medicine, James J. Peters VA Medical Center, New York, NY, United States; ^3^ Center for Translational Medicine and Pharmacology, Icahn School of Medicine at Mount Sinai, New York, NY, United States

**Keywords:** TSH (thyroid stimulating hormone), TSHR (thyroid-stimulating hormone receptor), neurite outgrowth, differentiation protocol, neurite length

## Abstract

Extra-thyroidal effects of TSH have been reported in various tissues expressing the TSH receptor (TSHR) including several areas of the brain. However, the influence of TSH on neuronal phenotypes has not been examined. Using a well-characterized human neuroblastoma cell line (SH-SY5Y), we have examined TSH signaling effects on the phenotype of these cells after their neuronal differentiation. Following an 18-day differentiation protocol, we successfully redifferentiated the SH-SY5Y cells into ~100% neuronal cells as indicated by the development of extensive neurofilaments with SMI-31 expression. Furthermore, using absolute digital PCR, we quantified TSHR mRNA, and also TSHR protein expression, in the redifferentiated cells and found that the neuronal cells expressed high quantities of both TSHR message and protein at baseline. Exposure to TSH induced primary, secondary, and tertiary neurite outgrowths, which are essential for cell–cell communication. Quantitative analysis of neurites using ImageJ showed a dose-dependent increase in neurites. The addition of TSH up to 1 mU/ml resulted in a ~2.5-fold increase in primary, and ~1.5-fold in secondary and tertiary neurites. The lengths of the neurites remained unaffected with the dosage of TSH treatment. Furthermore, TSHR signaling in the differentiated cells resulted in enhanced generation of cAMP, pPI3K, pAKT, and pNFkB pathways and suppression of pMAPK suggesting an influence of these signals in driving neurite outgrowth. These data showed that the TSH/TSHR axis in neurons may contribute to enhanced neurite outgrowth. The potential pathophysiological effects of TSH on the induction of neurite outgrowth and its relationship to neurodegenerative diseases remain to be explored.

## Introduction

The thyroid-stimulating hormone receptor (TSHR) is a critical human autoantigen in autoimmune thyroid disease and the major regulator of the thyroid cell (1, 2). Structurally, the TSHR is a G-protein-coupled receptor (GPCR) with seven transmembrane domains (TMD) and a large extracellular ectodomain (ECD), which is connected to the TMD via a flexible hinge (or linker) region (3). TSHR activation triggers multiple signaling pathways by engaging different G-proteins and β-arrestins that ultimately result in thyroid growth, thyrocyte differentiation, and the synthesis and secretion of thyroglobulin and thyroid hormones, including thyroxine (T4) and triiodothyronine (T3). However, recent studies have shown that TSHR expression is not confined to the thyroid but is present in a variety of cell types such as in the anterior pituitary gland (4), hypothalamus (5), gonads (6), bones (7), epidermis and hair follicles (8), kidney (9), immune system cells (10), white and brown adipose tissue (11), many different fibroblasts (12), and different brain regions (13). While the physiological roles in some of these extra thyroidal sites have been explored, others remain uncertain (6). For example, activation of TSHR and its signaling on white adipocytes have demonstrated its influence on adipogenesis, and the phenotype of these fat cells has been altered by knockdown of the TSHR (11). Furthermore, the TSHR is involved in the differentiation of pre-adipocytes into mature adipocytes derived from embryonic stem cells due to its influence on myriad signaling pathways (2). The activation of the TSHR appears to have connections with the regulation of food intake and seasonal reproductive patterns in the hypothalamus of birds (6), and in human periorbital tissue, it is associated with the regulation of orbital fibroblast differentiation and remodeling of the orbital tissue (14). Within the kidney, the TSHR plays a role in the regulation of renal development, morphology, and function (6), and in human bone marrow-derived mesenchymal stem cells, the TSHR is implicated in processes such as self-renewal, maintenance, and differentiation (6). Recently, we have uncovered widespread glycoprotein hormone receptor expression in mouse and human brains using RNAScope including the abundant presence of TSHR mRNA in specialized cells of the hypothalamus such as the tanycytes of the third ventricle (15). These and other variable effects of TSHRs in various tissues prompted us to ask what is the influence of TSHR activation on the neuronal phenotype.

To examine the influence of TSHR activation on the phenotype of neuronal cells, we used the well-characterized neuronal SH-SY5Y cell line, which has the ability to transit from a neuroblast-like state to a fully developed human neuronal cell under controlled differentiation conditions (16). These cells have a stable karyotype containing 47 chromosomes (17). First, we evaluated and characterized TSHR expression before and after differentiation and assessed the effects of activation of TSHR by TSH on neuronal characteristics including neurite outgrowth and neurite length. By evaluating various signaling molecules in these differentiated cells, we also showed that the expressed TSHRs were indeed functional.

## Materials and methods

### Cell culturing and treatments

SH-SY5Y cells were cultured in basic growth medium including Eagle’s Minimum Essential Medium (EMEM) (Sigma Cat#M4655), 15% hi-FBS (heat-inactivated fetal bovine serum) (LDP Cat#FBS-02 heat inactivated), 2 mM glutamine, and Pen/Strep and passaged every 3–5 days. For differentiation, cells were detached using Trypsin/EDTA 0.5% and cultured in the first differentiation medium containing EMEM, 2.5% FBS, 2 mM glutamine, 10 µM all-trans retinoic acid (ATRA) (Sigma Cat#R2625), and Pen/Strep. The cells were then moved to the second differentiation medium with 1% FBS. The cells were maintained in this medium for 3 days. Subsequently, the cells were transitioned into the third differentiation medium containing neurobasal medium, 1× B27, 20 mM potassium chloride (KCl), 2 mM GlutaMAX (Thermo Fisher Scientific Cat# 35050061), 50 ng/ml BDNF (brain-derived neurotrophic factor) (Sigma Cat# SRP3014), 2 mM dibutyryl-cAMP (db-cAMP) (Sigma Cat#28745-M), 10 µM ATRA, and Pen/Strep. In our modified protocol, we eliminated the initial ECM coating of the plates and observed better differentiation if we avoided the splitting of cells on day 7 and 10 of differentiation from previous existing protocols (17, 18). The occurrence of successful differentiation was confirmed by staining for SMI-31 with PhosphoDetect™ Anti-Neurofilament H mAb (called SMI-31 by Millipore Sigma Cat#NE1022), which is a cytoskeletal marker and used in the concentration of 1:1,000. Furthermore, for assessing the effects of TSH on neuronal characteristics, bovine thyroid stimulating hormone (TSH) was from Sigma (Cat# T8931) and supplemented in the medium in varying doses.

### Measurement of TSHR message levels by digital PCR

Since we have used differentiation protocol which can possibly alter the standard endogenous pool within the cells, we have used the absolute quantification of TSHR between undifferentiated and fully differentiated SH-SY5Y cells using the absolute digital PCR machine, which does not depend on the cycle threshold of the endogenous gene and the gene of interest. The data obtained by this method can be more reliable because the data reduced to negative and positive counts of the partitioned sample, thus eliminating assay efficiency and instrument dependent variability. The precision is controlled by the number of replicates done for each. In addition to comparing the data to that of the undifferentiated cells, we also used cells that overexpress the TSHR receptors as positive control (JPO9) (Chinese Hamster Ovary (CHO) overexpressing the human TSHR) (19). Total RNA was extracted using RNeasy Mini kit (Qiagen Ltd Cat#74134) in combination with ribonuclease-free deoxy-ribonuclease treatment to remove endogenous DNA contamination, and the concentration and quality of extracted RNA were measured by NanoDrop. 1 µg of total RNA was reverse transcribed into cDNA using RNA to cDNA EcoDry™ Premix (Double Primed) (Takara Bio, Cat# 639547). The PCR reaction for each sample contained 1.8 µl dPCR master mix (Thermo Fisher Scientific Cat# A52490), 0.45 µl PCR TSHR probe directed to the transmembrane region of the receptor (Thermo Fisher Cat number 4453320), and a fixed amount of cDNA samples, which were made to a final volume of 9 µl with DEPC water. The program was as follows: Cycle 1 Denaturation: 10“ at 96°C, Cycle 2 Amplification 5“ at 96° and 30“ at 60° 40×. Absolute digital PCR software uses Poisson statistics and presents the corrected data for each sample as copies/µl for each sample based on the threshold that is determined by the sample that is used as a reference, which shows anywhere 5%–85% negative wells in the partitioned matrix. We have used more than three replicates per sample, and data presented here are average of two such biological replicates.

### Immunostaining the TSH receptor

Confirmation for the existence of TSHR in neuronal cells and its localization in these neuronal cells was done with TSHR immunostaining using a TSHR-specific mouse monoclonal antibody MC1 directed against the hinge region of TSHR (epitope aa321-344) (20). Fisher rat thyroid cells (FRTL5) (which is a rat thyroid cell line) were used as the control. The FRTL-5 cells were grown in the six hormone medium (21) and were used as a positive control. For immunostaining, cells were cultured and differentiated in glass-bottom dishes at a density of 50,000 cells. After washing 1× with PBS pH 7.2, the cells were fixed with 4% paraformaldehyde in PBS at RT for 15 min. After fixation, cells were washed three times with PBS. The cells were blocked with 3% BSA and 5% horse serum in 1× PBS containing 0.3% Triton X-100 in the blocking buffer for 2 h. The fixed and blocked cells were treated with MC1 antibody (1:500), in a staining buffer containing 0.1% Tween 20 and 1% BSA in PBS overnight at 4°C. The next day, cells were washed three times with PBS and incubated with the anti-mouse conjugated to Alexa 488 (Cell Signaling Technology Cat# 4408) at 1:500 for 1 h at RT and then washed three times with PBS. Finally, the cells were mounted by mounting media containing DAPI (Vector Laboratories, Burlingame, CA) for nuclear staining. Fluorescence images were captured using ECLIPSE Ti2 inverted microscope (Nikon, Tokyo, Japan) equipped with NIS-Elements software (version 5.30.02, Nikon, Amsterdam, Netherlands) with 20×, 40×, and 100× objectives. ImageJ was used to adjust for brightness of all the images.

### Assessment of TSHR signaling by In-Cell Western

SH-SY5Y cells were grown in 96-well black bottom plates at 25,000 cells per well in the differentiation medium. After 18 days of differentiation, an assessment of cAMP for the functionality of TSHR in neuronal cells was performed using In-Cell Western, as described in our previous studies (22–24). Differentiated neuronal cells were treated with different doses of TSH (0, 1, 10, 100, and 1,000 micro-units per ml (µU/ml) for 24 h before the assessment of cAMP. PKA and PKC signaling molecules were also assessed using the same assay. To confirm if the effects were specific to TSH, blocking anti-TSHR-mAb (Ki-70) was used to reverse the observed effects. Ki-70 is a human TSHR blocking monoclonal antibody that binds to the leucine-rich repeat domain (LRD) region of the TSHR (25). The activation media were removed, and then the cells were fixed by adding 150 µl of fixation solution including 4% formaldehyde in 1× PBS for 20 min at room temperature. Cells were washed five times with 1× PBS containing 0.1% Triton X-100 (as a permeabilization agent) for 5 min each time, and 200 µl of washing solution was added and stayed for 5 min on the shaker and then removed; it was repeated four more times. Then, 50 µl of blocking buffer including BSA 5% in 1× PBS was added and stayed overnight at 4°. The blocking buffer was removed, and 25 µl of primary antibody diluted in the blocking buffer (1:100 dilution) was added to the wells and incubated overnight at 4° (primary antibodies were from Cell Signaling Technology, including Phospho-Akt (Ser473) (D9E) XP^®^ Rabbit mAb #4060, NF-κB p65 (D14E12) XP^®^ Rabbit mAb #8242, Phospho-PERK (Thr980) (16F8) Rabbit mAb #3179, and Phospho-PKCδ (Thr505) Antibody #9374). Cells were washed five times in 1× PBS containing 0.1% Tween 20 again five times each time for 5 min. Finally, 25 µl of infrared Fluorescent Dye (IRDye) subclass-specific antibody (IRDye 800CW LI-COR secondary antibody) in 1:800 dilution was added, in the dark for 60 min at room temperature on a shaker. Then, the cells were washed with PBS 1× containing 0.1% Tween 20 for five times and then visualized under the microscope.

### Quantitation of neurite count and length in TSH treated cultures

As described for immunostaining, the cells were grown in 35-mm glass-bottom dishes at a seeding number of cells of 50,000. After staining the nuclei with DAPI, phase images and DAPI-stained images were acquired as two independent image files under the same settings. DAPI-stained nuclei cell bodies and a phase image were applied to define the extension threshold for quantification. We used a plugin named AutoNeuriteJ in ImageJ and counted the number of neurite outgrowths and their lengths both for primary (first neurite out of the cell body) and secondary/tertiary (S/T) outgrowths (which are defined as the further extensions from the primary neurite) (26). The measurement was done on four different images of each TSH treatment (n=4), and the result is shown here as the mean ± SD.

## Results

### Establishment of neuronal phenotype from SH-SY5Y cells

**Figure 1:**
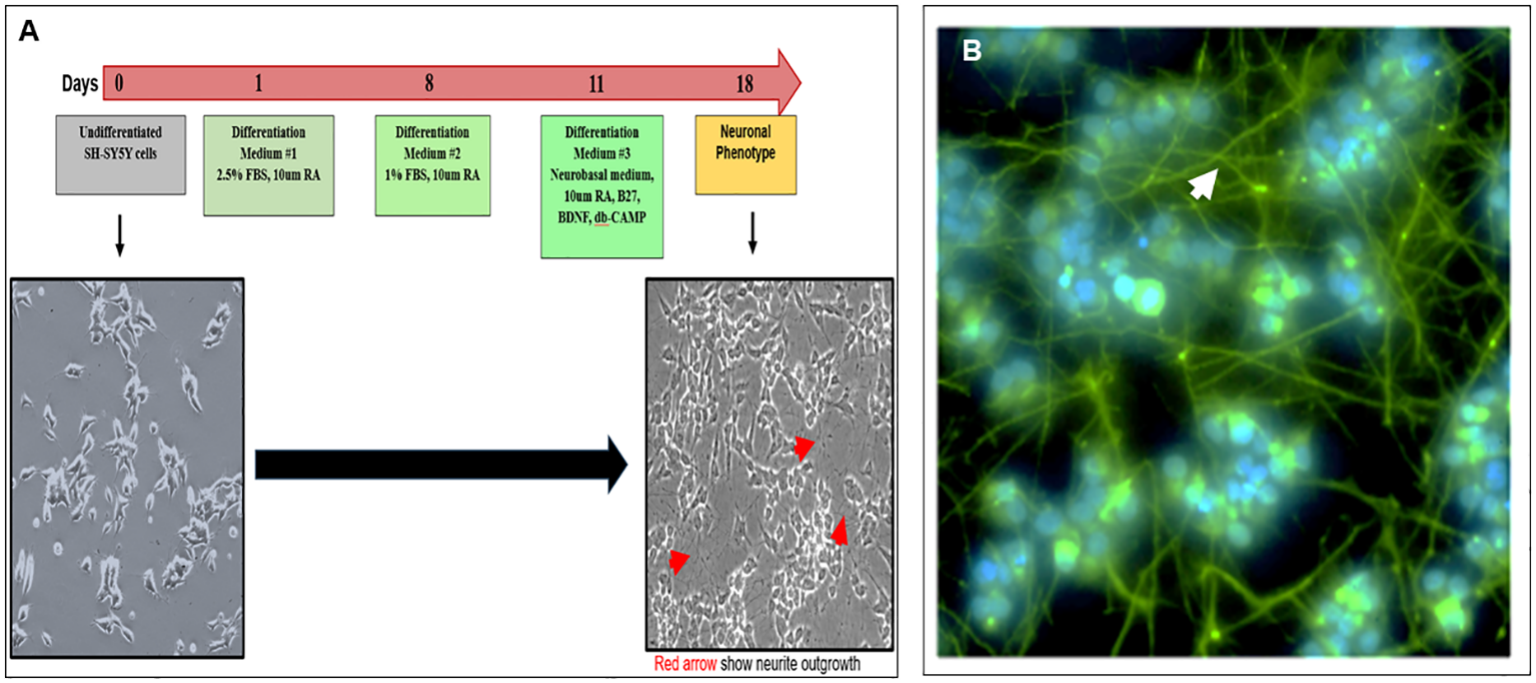
Modified protocol for differentiation of SH-SY5Y cells into neuronal cells **(A)** Diagram shows the redifferentiation protocol of making neuronal cells from undifferentiated SH-SY-5Y cells and phase-contrast images of the undifferentiated (left) and differentiated (right) neuronal cells (magnification ×20). **(B)** Confirmation of neuronal phenotype using an anti-SMI-31 labeled with secondary Alexa 488 showing extended neurofilaments and neurite outgrowths (magnification ×100).

Cultured SH-SY5Y neuroblastoma cells remained in an undifferentiated status with no neurite outgrowth. However, these cells were amenable to differentiation using an 18-day protocol as outlined in **Figure 1A**. We developed a modified differentiation protocol where the medium contained 15% hi-FBS with no retinoic acid (RA) from day 0. As shown in the schematic diagram, undifferentiated cells cultured in high serum were subsequently transferred into a differentiation medium in the presence of 10 µM of RA followed by sequential decreases in serum concentration to 1% by day 8. On day 11, these cells were further subjected to additional factors including B27, BDNF, and db-cAMP until day 18. On redifferentiation, these cells developed easily visible neurite extensions (**Figure 1A** phase image), which were visualized by staining their neurofilaments with antibody to SMI-31 shown by the green-stained axonal body and neurite outgrowth and blue DAPI-stained nuclei (**Figure 1B**) and demonstrated ~100% efficiency of differentiation into a neuronal phenotype.

### Assessment of TSHR expression in differentiated neuronal cells

**Figure 2:**
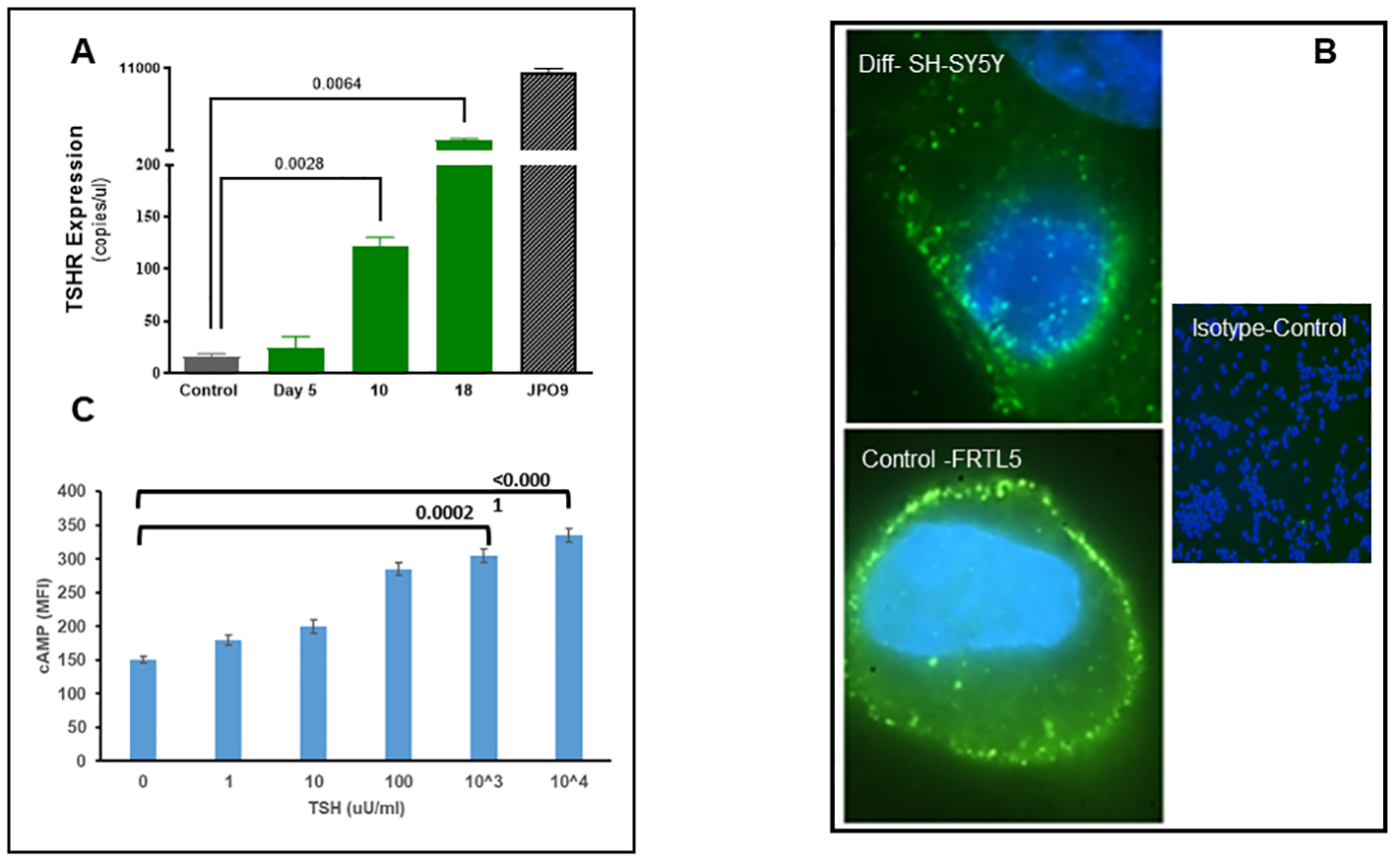
Expression and functional TSH receptors in differentiated SH-SY5Y cells. **(A)** TSHR was assessed by absolute digital PCR during different stages of differentiation and compared with the undifferentiated control cells. TSH expression was nearly 1,500 copies/µl on day 18 of differentiation when cells showed a neuronal phenotype. In comparison, TSHR expression in overexpressed JPO9 cells was 10,000 copies/µl. **(B)** The top image shows the expression of TSH receptors on the surface of the differentiated cells on day 18. TSHR expression was detected using a TSHR-specific monoclonal antibody against the hinge region (MC1). The bottom image is rat thyroid FRTL5 cells as the positive control, and the image on the right is the negative control (magnification 100×). **(C)** cAMP was measured after 1 h of stimulation of differentiated cells with bTSH using In-Cell Western. A dose-dependent increase of cAMP was observed, suggesting functional TSHRs. P-value <0.05 considered as significance. Data represent the mean ± standard error of the mean (SEM) in triplicate. MFI, mean fluorescence intensity.

We evaluated the expression of the TSHR in the differentiated cells at the mRNA and protein levels. Message levels of the TSHR were assessed by quantification of the absolute copy numbers of TSHR in undifferentiated versus differentiated cells using digital PCR (ddPCR). TSHR mRNA levels were quantified after 5, 10, and 18 days of differentiation. This time course assessment showed a significant increase in TSHR mRNA by day 18 and reached over 1,500 copies from a baseline of ~20 copies (**Figure 2A**). TSHR protein was detected by immunostaining with a TSHR-specific monoclonal antibody against the hinge region of the TSHR (MC-1) (20). As can be seen in **Figure 2B**, there was a surface expression of receptor protein in the differentiated cells. Optical sectioning of the stained cells showed the distribution of the TSHR expression throughout the surface of the differentiated cells (**Figure 2B** upper).

### TSHR signaling by redifferentiated neuronal cells

**Figure 3:**
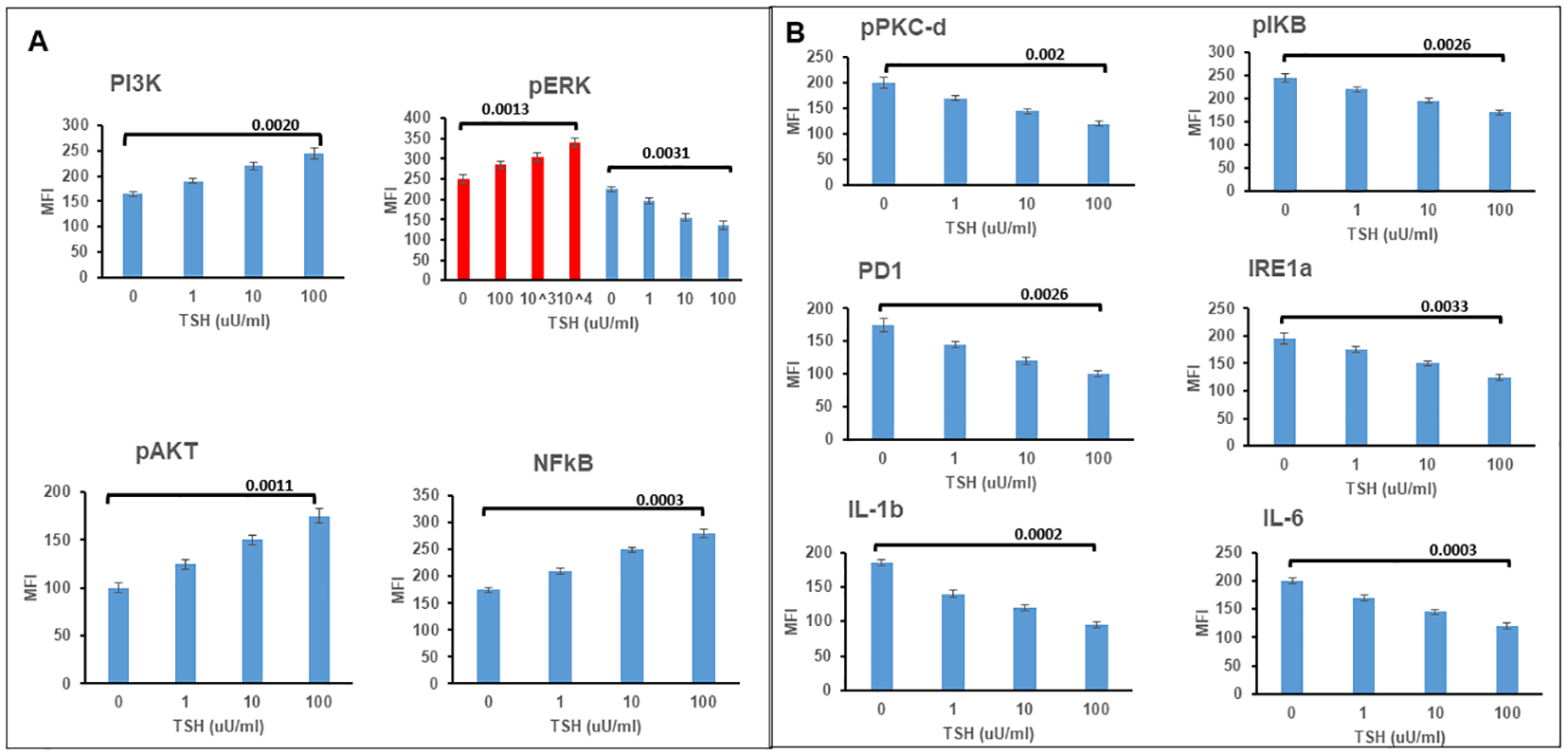
TSH induced non-classical signaling in differentiated SH-SY5Y cells. **(A)** Assessment of classical TSHR signals. Note the increases at 24 h in P13K, pAKT, and NFkB. pERK showed an increase by 2 h, as shown in red and then the response was lost. **(B)** Non-classical signals in differentiated cells also after TSH treatment showed decreases rather than increased responsiveness. Data represent the mean ± standard error of the mean (SEM) in triplicate. MFI, mean fluorescence intensity.

Cyclic AMP generation by the differentiated SH-SY5Y cells after stimulation with TSH (**Figure 2C**) showed a dose-dependent increase, suggesting the presence of functional receptors. Since the TSHR is known to engage with different G proteins and beta arrestins for downstream signaling effects on cell proliferation and survival, we assessed other classical signals including pPI3K, pERK, p AKT, and NFKB and non-classical signals including pIKB, IRE1a, IL6, and PD1, on stimulation with increasing concentrations of TSH. Our previous studies have shown that activation of the TSHR with TSH or with stimulating TSHR autoantibodies leads to an array of signal pathway activity (23, 27, 28). Using TSH in a dose-response from 1 µU/ml to 1,000 µU/ml over 24 h, we measured these effects by In-Cell Western assays (29) and found AKT, NFkB, and PI3K signals in these cells increased in a dose-dependent manner (**Figure 3A**), confirming that some of the expected classical signals in addition to cAMP were increased by activation of the TSHR in these neuronal cells. In contrast, the non-classical responses were dampened rather than exacerbated (**Figure 3B**) as also seen with pERK, which after showing an early response was then suppressed (**Figure 3A**).

### TSH initiates and enhances primary and secondary/tertiary (S/T) neurite outgrowth

**Figure 4:**
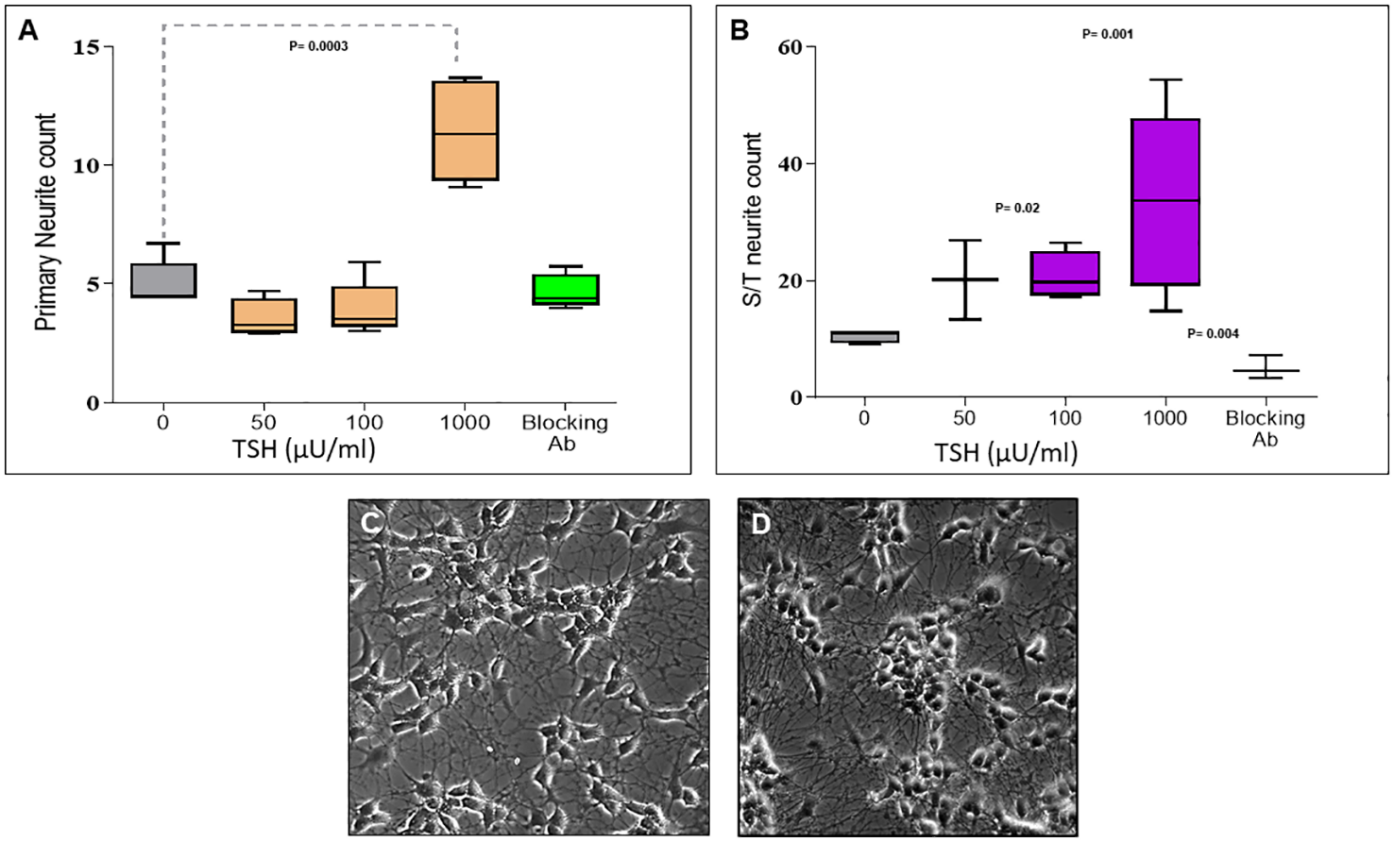
Dose-dependent effect of TSH on neurite outgrowth. **(A)** The effect of TSH treatment over 18 days on the primary neurite counts, which increased with high concentrations of TSH. The effect was lost in the presence of a blocking anti-TSHR-mAb (Ki-70). **(B)** The effect of TSH treatment on secondary and tertiary (S/T) neurite counts. bTSH was supplemented in every media change during differentiation, and again its effect was blocked by anti-TSHR-mAb Ki-70. P-value <0.05 considered as significance. Data represent mean ± SD for four different images of each TSH treatment. **(C)** One of the representative images which was analyzed for neurite count and length at the same time, which is neuronal cells after 18 days of differentiation. **(D)** One of the representative images which was analyzed for neurite count and length at the same time, which is neuronal cells after 18 days of differentiation supplemented with 100 µU bTSH.

**Figure 5:**
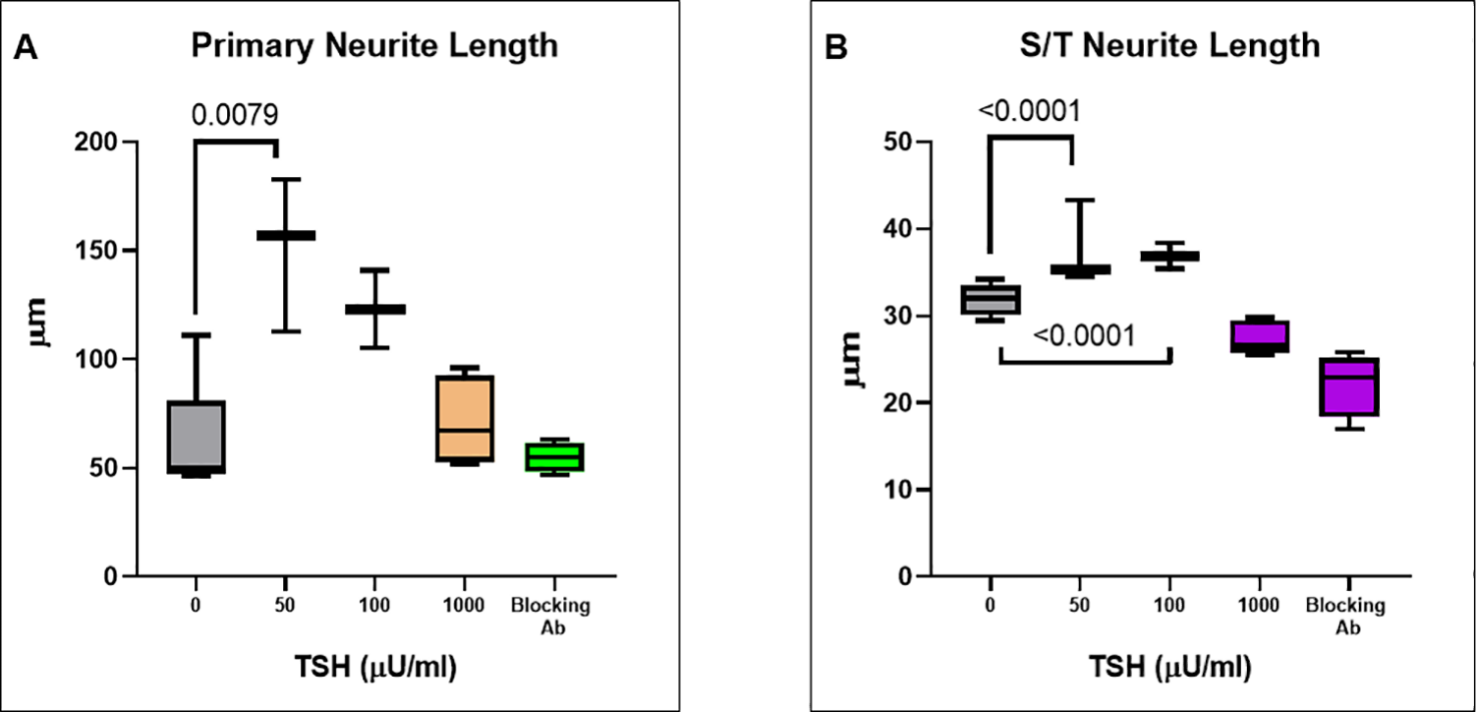
TSH-induced changes in neurite length. **(A)** The effect of TSH treatment over 18 days on the primary neurite length which also increased with TSH. The effect was lost in the presence of a blocking anti-TSHR-mAb (Ki-70). **(B)** The effect of TSH treatment on secondary and tertiary (S/T) neurite length. bTSH was supplemented in every media change during differentiation and again its effect was blocked by anti-TSHR-mAb Ki-70. P-value <0.05 considered as significance. Data represent. Mean ± SD for four different images of each TSH treatment.

Having observed functional TSHRs on the differentiated neuronal cells, we then examined the effect of TSH on neurite outgrowth. The cells were supplemented at each differentiation medium change, with 50 µU/ml, 100 µU/ml, or 1000 µU/ml of TSH, and neurite growth was found to be stimulated by increasing concentrations of TSH (**Figure 4A**). The primary neurite outgrowth count showed a ~2.5-fold increase, and this effect of TSH was effectively blocked by pretreating the cells with a human TSHR blocking antibody (K1-70, 10 ug/ml). Similarly, quantification of S/T neurite outgrowth also showed a ~1.5-fold increase (**Figure 4B**). **Figures 4C, D** displays representative images analyzed for neurite count and neurite length in both untreated and TSH-treated cells. These results confirmed that TSH enhanced neurite outgrowth in these neuronal cells. For neurite length, in a low dose of TSH, a significant difference was observed, which did not show a dose response (**Figure 5**).

## Discussion

The long-held concept of glycoprotein hormones having narrowly confined targets has been usurped by the mounting evidence for their disparate expression and actions in non-pituitary locations (6). The anterior pituitary hormones are advanced examples of this concept, and none is a better example than the thyroid-stimulating hormone (TSH), which has been shown to have the expression of its functional receptor (TSHR) in multiple organs and tissues (6). While the TSHR expression in the brain has been known for some time (13) and its role in a variety of neuronal activities has been documented (6), we have recently mapped both mouse and human brain TSHR expression to begin to understand those specific areas of the brain that may show TSHR activity and how the receptor and its ligand and various molecules that target the TSHR may influence neuronal phenotype and function (15). The areas with extensive neuronal TSHR expression include the third ventricle and tanycyte cells lining the third ventricle in the hypothalamus, which has extensive connections with known TSH and thyroid hormone action (30).

In order to show that a neuronal TSHR has an influence on its phenotype, we needed to first examine its potential in a model cell system. For these studies, we chose a well-characterized human neuroblastoma cell line (SH-SY5Y), which could be redifferentiated into neuronal cells (31) with many characteristics of normal cells. We found that such undifferentiated cells developed well under modified differentiating conditions and expressed the specific neuronal cytoskeletal marker SMI-31, characteristic of mostly differentiated neurons, and then grew to form rudimentary neurite networks. This has proved a useful model for examining the functional attributes of modifying ligands such as TSH.

Interestingly, the TSHR transcript levels increased >200-fold as the cells fully differentiated on day 18 (**Figure 2A**). However, such increase cannot be compared with data presented by RNAScope (15) in different regions of the brain because expression patterns can vary a lot between different neuronal types as seen by variability of expression between different regions of the brain. We can only say with certainty that in such monoculture, we see an increase TSHR expression compared with day 0 cells. Increased TSHR expression in extra-thyroidal locations has previously been linked to differentiation. For example, studies have shown that TSHR expression increases as pre-adipocytes differentiate into adipocytes (32). The mechanism for such an increase can be related to various factors, one of them being increased promoter activity leading to increased mRNA transcription. The signals and coactivators leading to such an increase in TSHR transcription remains to be defined. TSHR protein expression on the surface of the cells as shown by immunostaining with TSHR-specific monoclonal antibody (**Figure 2B**) only suggests that these differentiated neurons do not merely make the transcripts but do possess the translational capability and the right trafficking machinery to express the receptors on the surface as found in rat thyroid.

TSHR transcripts expressed on the cell surface were indeed functional TSHRs with signaling ability, suggesting a functional role for the receptors in neuronal cells. Evidence for this signaling was abundant and appeared to include the classical pathways as described for the thyroid TSHR (33). Since the TSHR is constitutively active (34), it may have a functional state that is ligand independent at baseline in addition to being under TSH regulation. At this stage, we only know that TSH can signal via these functional neuronal TSHRs and that activation of such signaling influences neurite outgrowth demonstrating the neuritogenic activity of TSH. Since neuritogenesis is a characteristic of neuro-regeneration and that elongations of axons and dendritic arborization are needed to receive and send signals between cells and other parts of the brain, the growth of neurites induced by TSH would have a significant impact on neuronal communication. Our study has shown that activation of TSHR by TSH has a role in enhancing the phenotype of these cells via neurite outgrowth. Although our signal profiling studies outlined here did not pinpoint the exact signals that are responsible for this action of TSH, the fact that we see MAPK pathway signals such as ERK suppressed and cAMP and other classical signals such as PI3K increased would suggest that these signal feeds have a role in neuritogenesis, which is akin to similar signaling mechanisms proposed for the nerve growth factor (NGF) on neurite growth (35).

A limitation of this study is represented by the fact that our findings and conclusions are based on observations obtained from a single neuroblastoma-derived cell line. This limitation should be considered when extrapolating the results to more diverse neuronal systems or human physiology. Further studies using additional cell types or *in vivo* models would help clarify the broader implications of TSHR modulation in the nervous system.

Considering that *in vitro*, 1 uU/ml of bovine TSH has almost no activity in any cell or assay system since studies of TSH began and that in humans TSH levels can reach more than 100 uU/ml in hypothyroidism and in the older population TSH levels are 5 uU/ml–10 uU/ml, highlights the need for further research to explore the impact of TSH at more physiologically representative concentrations.

Hence, the findings of our study have shown that TSHR modulation by the TSH ligand induces neurite outgrowth, suggesting broader implications in neuronal regeneration, and such mechanisms can be useful in understanding pathologies of the nervous system where there is retraction of neurites due to pathological conditions or damage. This action of TSH via the TSHR can also be a useful approach to reconstruction of neuronal and synaptic networks in the brain. Furthermore, abnormal TSH levels have been associated with a variety of pathologies including myxedema madness (36) and Parkinson’s disease (37), and such associations may not be exclusively thyroid hormone related but rather the thyroid hormone independent action of TSH. TSH effects could be a double-edged sword in neuronal cells.

## Data Availability

The original contributions presented in the study are included in the article/supplementary material. Further inquiries can be directed to the corresponding author.
